# Protein C receptor maintains cancer stem cell properties via activating lipid synthesis in nasopharyngeal carcinoma

**DOI:** 10.1038/s41392-021-00866-z

**Published:** 2022-02-16

**Authors:** Panpan Zhang, Qiuping He, Yaqin Wang, Guanqun Zhou, Yupei Chen, Linglong Tang, Yuan Zhang, Xiaohong Hong, Yanping Mao, Qingmei He, Xiaojing Yang, Na Liu, Jun Ma

**Affiliations:** 1 Department of Radiation Oncology, Sun Yat-sen University Cancer Center, State Key Laboratory of Oncology in South China, Collaborative Innovation Center of Cancer Medicine, Guangdong Key Laboratory of Nasopharyngeal Carcinoma Diagnosis and Therapy, Guangzhou, 510060 China; 2 Max-Planck Center for Tissue Stem cell Research and Regenerative Medicine, Bioland Laboratory (Guangzhou Regenerative Medicine and Health Guangdong Laboratory), Guangzhou, 510530 People’s Republic of China

**Keywords:** Cancer stem cells, Head and neck cancer

## Abstract

Metastasis and recurrence account for 95% of deaths from nasopharyngeal carcinoma (NPC). Cancer stem cells (CSCs) are regarded as one of the main reasons for tumor cell resistance to clinical therapy, and cancer metastasis or recurrence, while little is known about CSCs in NPC. The present study uncovers a subpopulation of cells labeled as CD45^−^EPCAM^+^PROCR^+^ in NPC biopsy samples that exhibit stem cell-like characteristics. A relatively low number of these cells initiate xenograft tumors in mice. Functional analysis reveals that protein C receptor (PROCR) not only serves as a stem cell marker in NPC, but also maintains tumor cells’ stemness potential through regulating lipid metabolism and mitochondrial fission. Epistatic studies reveal that cAMP-protein kinase A stimulates Ca2^+^ release to manipulate lipid metabolism related genes’ expression. Finally, in a cohort of 207 NPC samples, PROCR expression is correlated with tumor metastasis or recurrence, and predicts poor prognosis. These novel findings link PROCR labeled CSCs with lipid metabolism and mitochondrial plasticity, and provides new clinical target against metastatic or recurrent NPC.

## Introduction

Nasopharyngeal carcinoma (NPC) is a highly malignant head and neck cancer with regional aggregation in South China, Southeast Asia, North Africa, the Mediterranean basin, and Alaska.^1–4^ Distant metastasis and recurrence account for the main cause of death in NPC, and remain as the bottleneck to improve the cure rate of NPC in the clinic. Complicating matters further, the mechanisms underlying these symptoms still remain obscure. Cancer stem cells (CSCs) are speculated to be the root cause of distant metastasis and recurrence.^5–7^ However, no confirmed CSCs have been hitherto reported yet in NPC. Efforts to identify CSCs will provide the possibility to cure metastatic or recurrent NPC using more targeted clinical strategies.

Endogenous lipids are classified into triglycerides, sterols, phosphoglycerides, and sphingolipids. Triglycerides, synthesized from fatty acids, are mainly used for energy storage together with cholesterol in the form of lipid droplets in organisms. In response to the massive demand of macromolecular and bioenergy, cancer cell reprograms metabolism to support their initiation, proliferation, migration, and survival.^8^ Therefore, abnormality of lipid metabolism is thought to be associated with the development and deterioration of many cancers.^9,10^ It has been realized that lipid metabolism disorders contribute to a series of cellular components or processes alteration, including membrane, energy homeostasis, signal transduction, and hormone production. Typically, a common feature in cancer is the upregulation of lipogenesis and accumulation of lipid droplets partially in response to the demand of producing membrane for proliferation.^11–13^ Additionally, hyperactive lipid metabolism also contributes to the maintenance or survival of CSCs in some types of tumors.^14–16^ However, it remains obscure whether and how the lipid metabolism disorders are involved in NPC progression.

Protein C receptor (PROCR), also called endothelial protein C receptor (EPCR), is well known for its role in mediating anticoagulation or anti-inflammatory processes, triggered by its ligand, activated protein C (APC), together with protease-activated receptor-1 (PAR1).^17,18^ Recent studies revealed that PROCR also serves as a stem cell marker in the mammary gland,^19^ and as a CSC marker in triple-negative breast cancer.^20^ Activated PROCR is believed to participate in Ca^2+^ flux in human brain endothelial cells,^21^ the MAPK/ERK kinase 1 (MEK)-extracellular regulated kinase (ERK) signaling cascade in endothelial cells,^20,22^ and the Ras homolog family member A (RhoA)- Rho associated coiled-coil containing protein kinase (ROCK)-p38 pathway in breast cancer cells.^20^ However, little is known about PROCR in NPC. In the present study, we show that PROCR serves as a CSC marker, and ectopic expression of PROCR confers the stemness potential on NPC cells. Further functional analysis revealed that lipid metabolism and the associated mitochondrial fission are indispensable for tumor cells’ maintenance of stemness induced by PROCR. Collectively, these findings provide theoretical therapeutic targets to treat metastatic or recurrent NPC.

## Results

### Identification of an NPC cell group with stem cell-like properties

**Fig. 1:**
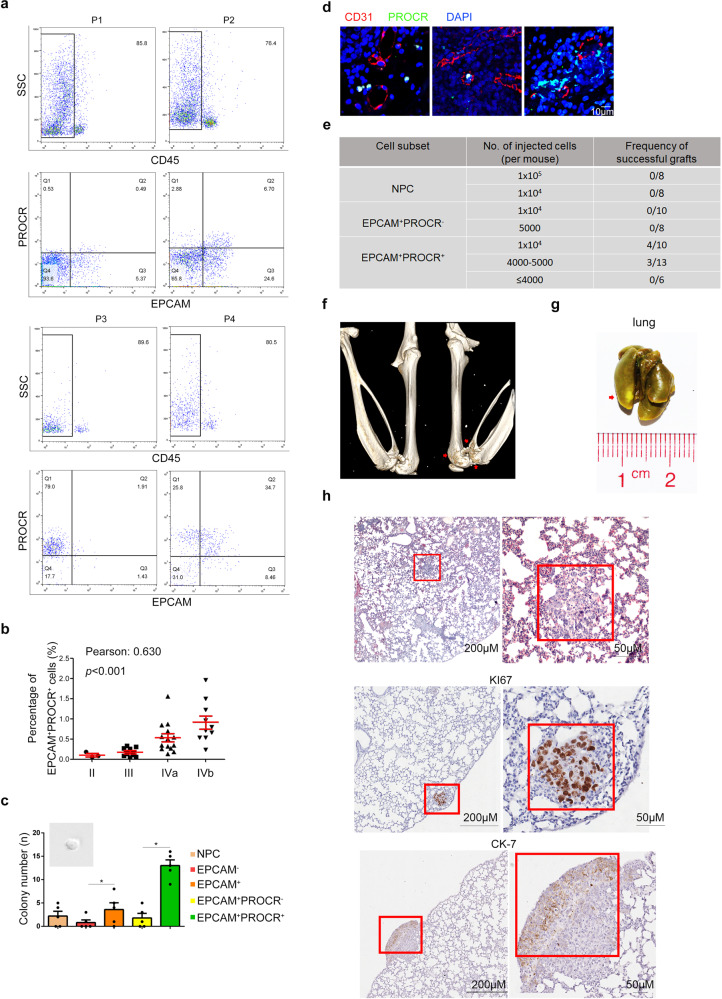
Identification of an NPC cell group with stem cell-like properties. **a** Flow cytometry analysis of CD45^-^EPCAM^+^PROCR^+^ cells in different patients with NPC. **b** Correlation analysis of the amount of CD45^-^EPCAM^+^PROCR^+^ cells with the matching TNM stages of the patients (*n* = 51). **c** Sphere formation assay of the sorted cells from NPC biopsy samples. All the sorted cells were gated from the CD45^−^ population. *P < 0.05. **d** Immunofluorescence staining of CD31 and PROCR in sectioned paraffin embedded NPC biopsy samples. **e** Summary of the xenograft results showing the number of sorted cells transplanted into the mice, and their corresponding engraftment efficiency. The successfully engrafted tumor was regarded as the ones with both osteolytic lesion and lung nodules in mice. All of the successfully engrafted specimens from different patients were pooled together. The CSC frequency is estimated to be 1/17152 according to Extreme Limiting Dilution Analysis (ELDA) with 0.95 confidence interval. **f** CT scanning of the mice femurs. The image shows the three-dimensional volume reconstruction of leg bones of a representative recipient mouse. The arrowheads label the osteolytic lesions resulting from tumor metastasis. **g** Lung metastases of the recipient mouse. The arrowheads mark the nodules in the lung, after incubating in the Bousin solution. **h** HE staining and KI67 expression of xenograft tumors in mouse lungs sections. The right panels show the red box regions at a higher magnification

In our attempt to identify CSC markers in NPC, we compared biopsy samples from NPC patients at different tumor-node-metastasis (TNM) stages, and found that the epithelial cell adhesion molecule (EPCAM)^+^PROCR^+^ cell population could be distinguished from other cell subtypes by their overrepresentation in specimens with advanced disease stage (Fig. 1a). Further analysis in a cohort of 51 patients showed that the percentage of CD45^−^EPCAM^+^PROCR^+^ cells (ranging from 0.1–2.0%) correlated with the patients’ TNM stage (Fig. 1b), suggesting that this cell group might play an essential role during NPC progression. To identify whether PROCR serve as a stem cell marker in NPC, we examined the self-renewal potential of EPCAM^+^PROCR^+^ cells through a sphere formation assay. The results showed that EPCAM^+^PROCR^+^ cells generated more and larger tumorspheres (Supplementary Fig. S1a, Fig. 1c). Next, we detected the existence of this cell group in the peripheral blood (PB) of patients with NPC and revealed that the amount of these cells correlated with their proportion in the primary lesion (Supplementary Fig. 1b, c). Moreover, in situ immunofluorescence exhibited that PROCR^+^ cells were positioned adjacent to CD31^+^ vessels (Fig. 1d), indicative of their metastatic potential. The sphere formation assay verified the self-renewal potential of the cells isolated from PB (Supplementary Fig. 1d), and the expression of canonical stem cell markers was also elevated in EPCAM^+^PROCR^+^ cells (Supplementary Fig. 1e). Therefore, we proposed that the EPCAM^+^PROCR^+^ population were potential CSCs.

Next, we determined the tumor initiating and metastatic potential of this cell population through the patient-derived xenograft (PDX) model using immunocompromised B-NDG (NOD-*Prkdc*^*scid*^*IL2rg*^*tm1*^*/*Bcgen) mice. Anatomically, mice lack the nasopharynx. Therefore, the different tumor cell subtypes were transplanted into the femoral medullar cavity of the mice, because it was demonstrated previously that the bone marrow serves as a reservoir or shield for disseminating tumor cells.^23–25^ Three to ten months later, the recipient mice were examined for xenograft tumor progression. It was observed that only EPCAM^+^PROCR^+^ cells generated successful xenografts (Fig. 1e, Supplementary Fig. 1g, and Table 1), and the mice displayed apparent osteolytic lesions in their femurs and nodules in their lungs (Fig. 1f, g). Moreover, human PROCR^+^ cells could also be detected in the PB of the recipient mice (Supplementary Fig. 1f), indicative of their metastatic potential. The results of hematoxylin and eosin (HE) staining, KI67 and CK-7 expression further validated that the nodules in the lung were differentiated human tumor cells (Fig. 1h). Thus, it can be concluded that PROCR labeled a cell population with CSCs property in NPC, which influenced the tumor initiation and metastasis.

### PROCR plays an important role in cancer stem cell in NPC

**Fig. 2:**
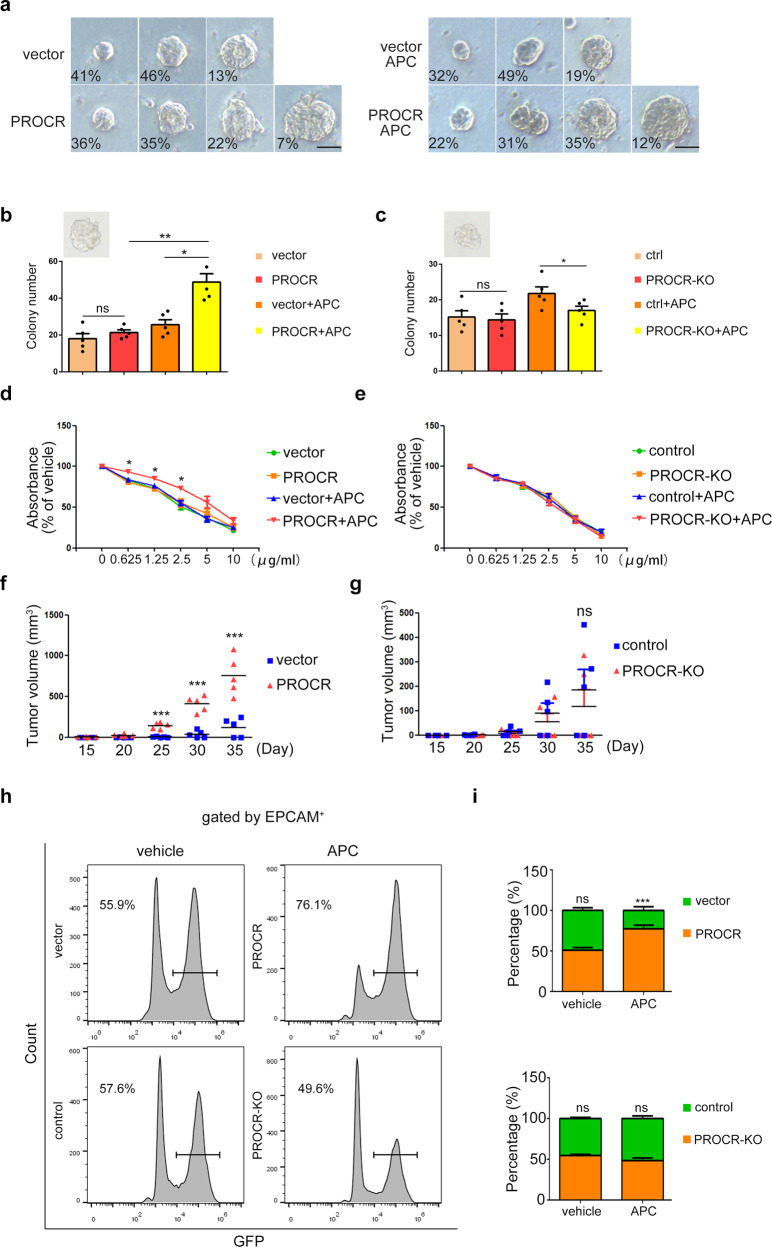
PROCR labels potential cancer stem cell in NPC. **a** Various tumorspheres distribution formed by 5–8 F cells. The scale bar denotes 50 µm. **b**, **c** Quantification of the tumorspheres with *PROCR* overexpression or knockout. The eligible tumor sphere is screened with a diameter greater than 50 µm. **d**, **e** CCK-8 assay of the *PROCR* overexpression or knockout cells with DDP treatment at different dosages. **f**, **g** Growth curves of the volume of subcutaneous xenograft tumors; *n* = 5 for each group. A total of 1 × 10^6^ tumor cells together with APC (300 μmol) were diluted into 200 μl phosphate-buffered saline (PBS) solution for subcutaneous transplantation. From 2 to 4 weeks, the APC (300 μmol) were treated by tail vein injection. **h**, **i** Flow cytometry analysis of the GFP^+^ cells from xenograft tumors in mice lungs, and their corresponding statistical analysis. APC was treated by tail vein injection. ns, not significant, *P < 0.05, **P < 0.01, ***P < 0.001

**Fig. 3:**
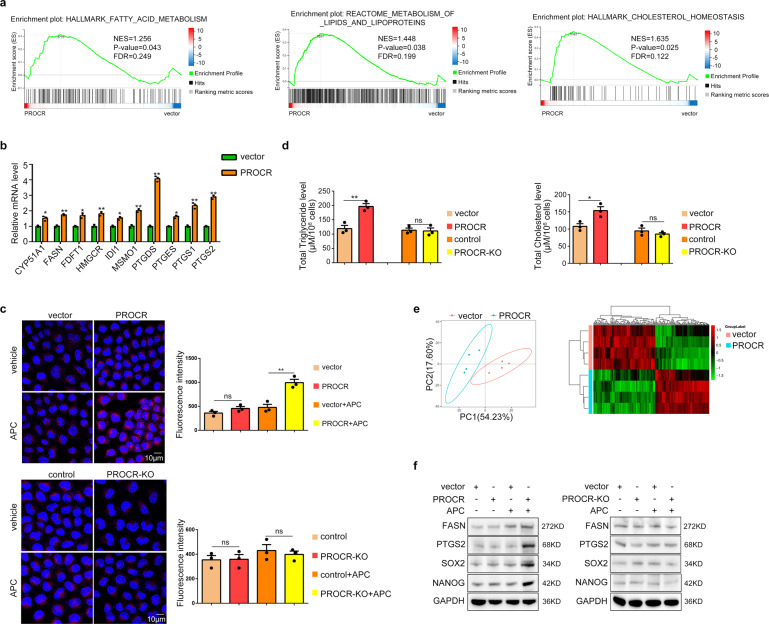
Lipid metabolism is aberrant in NPC cells with excessive PROCR activation. **a** GSEA analysis of the RNA-seq results comparing PROCR overexpressing cells and their counterpart control cells. The cells were harvested for RNA-seq after APC treatment. **b** mRNA expression of the lipid metabolism related genes with top ranked in the RNA-seq data by RT-qPCR. **c** Nile red staining of the cells with *PROCR* overexpression or knockout. The right panel shows the corresponding statistical analysis of the fluorescence intensity. **d** The cell content of triglycerides and cholesterol in *PROCR* overexpression or knockout cells. All of the cells were harvested after 48 h of APC treatment. **e** Left panel showed the PCA analysis of vector and PROCR GOF cell groups. Each group contains four samples. All of the samples were pretreated with APC. Right panel showed the differential lipid molecules analysis. Each column in the figure represents a difference ion, and each row represents a sample. Different colors indicate different intensities. **f** Western blotting analysis of lipid-related genes or stem cell markers expression in *PROCR* overexpression or knockout cells. ns, not significant, *P < 0.05, **P < 0.01

We then asked whether PROCR acts as a functional gene in tumor cells. Previous data comparing gene expression in NPC samples with normal nasopharyngeal tissues suggested that PROCR might function as an oncogene in NPC (Supplementary Fig. 2a), which was also supported by our RT-qPCR examination in various NPC cell lines (Supplementary Fig. 2b). To confirm this, we next overexpressed *PROCR* stably in 5–8 F cells, and knocked out *PROCR* in HONE1 cells using the clustered regularly interspaced short palindromic repeats (CRISPR) method (Supplementary Fig. 2c). Cell functional analysis revealed that PROCR gain-of-function (GOF) or loss-of-function (LOF) did not influence tumor cell proliferation, while GOF fueled cell’s invasiveness, migration, and colony-forming ability, and the effects were strengthened after APC treatment (Supplementary Fig. 2d–i). The subcutaneous xenograft tumor assay using PROCR GOF cells revealed that the tumor growth-promoting effect of PROCR was dependent on exogeneous APC treatment. Moreover, the sphere forming capability of PROCR GOF cells was significantly enhanced, while PROCR LOF cells exhibited no such effect (Fig. 2a–c). In addition, PROCR GOF enhanced the tumor cells’ resistance to the chemotherapeutic drug cisplatin (DDP) (Fig. 2d, e), validating the hypothesis that PROCR transformed tumor cells into candidate CSCs. Meanwhile, the protein expression level of stem cell markers SRY-box transcription factor two (SOX2) and NANOG were also elevated upon PROCR activation (Fig. 3e, Supplementary Fig. 5b). Next, we evaluated their stemness potential in mice. It was found that as low as ten cells with PROCR GOF could produce successful subcutaneous xenograft tumors (Supplementary Fig. 3a, Fig. 2f, g), indicative of a better self-renewal or tumor initiating capacity. To better determine the tumor metastatic potential, GFP was stably expressed in PROCR GOF or LOF cells, respectively, and transplanted into mice femurs after mixing with corresponding counterpart control cells. Four weeks later, the mice were sacrificed and the lungs with nodules were evaluated (Fig. 2h). The results showed that GFP^+^ cells accounted for the majority of tumor xenografts in the lungs in the presence of *PROCR* overexpression, but not in the *PROCR* knockout cells (Fig. 2h, i, Supplementary Fig. 3b, c). Therefore, PROCR endowed the tumor cells with stem cell-like properties, which increased their self-renewal and metastatic potential.

### Lipid metabolism is aberrant in NPC cells with excessive PROCR activation

To determine the molecular mechanism underlying PROCR’s effects, we used PROCR GOF 5–8 F and PROCR LOF HONE1 cells for RNA-sequencing. Facilitated by Gene Set Enrichment Analysis (GSEA), we revealed that genes related to fatty acids or cholesterol metabolism were enriched in GOF cells (Fig. 3a). In addition, Kyoto Encyclopedia of Genes and Genomes (KEGG) pathway analysis showed that the pathways relevant to lipid metabolism were upregulated upon *PROCR* overexpression (Supplementary Fig. 4a). We then examined the mRNA expression of the predicted genes involved in lipid metabolism, and the results supported our findings (Fig. 3b). Lipid droplet, which is composed of fatty acid and cholesterol, was labeled by Nile red in tumor cells. We found that PROCR GOF cells accumulated more lipid droplets in the presence of APC (Fig. 3c). Additionally, the endogenous concentration of triglycerides and cholesterol was also increased in GOF cells (Fig. 3d), confirming that activated PROCR accelerated lipid biosynthesis and accumulation in NPC. To reveal more details of lipid metabolism alteration, we also performed lipidomics using vector and PROCR GOF cells (Fig. 3e). The results revealed that PROCR overexpression remodeled the constitution of lipid metabolites (Fig. 3e), and the content of 102 lipid molecules increased while 89 decreased in PROCR GOF cells, of which fatty acid or steroid were all upregulated (Supplementary Fig. 4b), consistent with our RNA-Seq results.

Fatty acid synthase (FASN) and prostaglandin-endoperoxide synthase 2 (PTGS2) are the rate-limiting enzymes governing fatty acid *de novo* synthesis and prostaglandin E2 (PGE2) synthesis, respectively. As expected, their protein expression levels increased in PROCR GOF cells (Fig. 3f). Inhibition of these enzymes using antagonists or gene silencing by short hairpin RNAs (shRNAs) reduced the storage of lipid droplets or PGE2, respectively (Supplementary Fig. 4c–f).

### Inhibition of FASN or PTGS1/2 suppresses PROCR-induced stem cell potential

To investigate whether the PROCR-induced stem cell properties were dependent on aberrant lipid metabolism, we suppressed FASN or PTGS1/2 activity in activated PROCR GOF cells. The results showed that the mRNA or protein expression levels of SOX2 and NANOG were attenuated in PROCR overexpressing cells (Supplementary Fig. 5a, b). We next inhibited endogenous FASN or PTGS1/2 activity in PROCR^+^ NPC biopsy samples, and observed decreased expression of stem cell markers and attenuated sphere forming capability (Supplementary Fig. 5c, d). Additionally, the enhanced sphere forming and metastatic capability of PROCR GOF cells was reversed upon FASN or PTGS1/2 blockage (Supplementary Fig. 5e, f). To identify the influence of lipid metabolism on tumor initiating potential in vivo, we administered FASN or PTGS1/2 inhibitors to mice and evaluated xenograft tumor growth regularly. The results showed that the growth rate of subcutaneous xenograft tumor cells with *PROCR* overexpression was suppressed when FASN or PTGS1/2 were inhibited (Supplementary Fig. 5g). Then, the PROCR-GFP cells were mixed with the vector control cells and injected into mice femurs, followed by evaluation of the metastatic cells in the lungs. The results revealed that blockage of FASN or PTGS1/2 attenuated the competitiveness of PROCR GOF cells during metastasis (Supplementary Fig. 5h). Overall, these results showed that lipid metabolism is indispensable for NPC cells to maintain their stem cell-like properties.

**Fig. 4:**
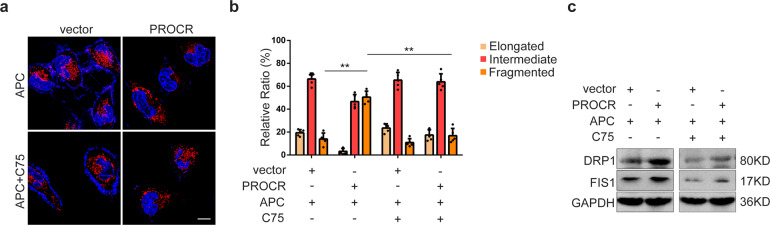
PROCR triggers mitochondrial fission through activating lipid synthesis. **a** Mitochondrial dynamics labeled by MitoTracker staining in tumor cells with APC or C75 treatment. C75, FASN inhibitor. Scale bar denotes 10 µm. **b** Quantification of the proportion of mitochondria with three different statuses. The relative number of mitochondria with different statuses was counted from about 100 cells in each treatment group. **P < 0.01. **c** Western blot examination of genes expression essential for mitochondrial fission in NPC cells with different treatment

Enlightened by previous findings reporting lipid metabolism correlated with mitochondrial dynamics in stem cells,^26–28^ we ask whether mitochondrial dynamics were involved in PROCR mediated tumor stem cell maintenance in NPC. Intriguingly, mitotracker labeling revealed that PROCR GOF cells displayed more fragmented mitochondria (Fig. 4a, b). In addition, DRP1 and FIS1, the two essential regulators during the mitochondrial fission process, were elevated upon *PROCR* overexpression, while decreased after inhibition of lipid synthesis (Fig. 4c). Additionally, the expression of mitochondrial fusion marker MFN1 decreased, while mitochondria content protein UQCRC1 and COX4I1 remained unaltered (Supplementary Fig. 5i), supporting that PROCR stimulated mitochondrial fission, and the process was dependent on lipid synthesis.

### Signaling cascade triggered by PROCR

**Fig. 5:**
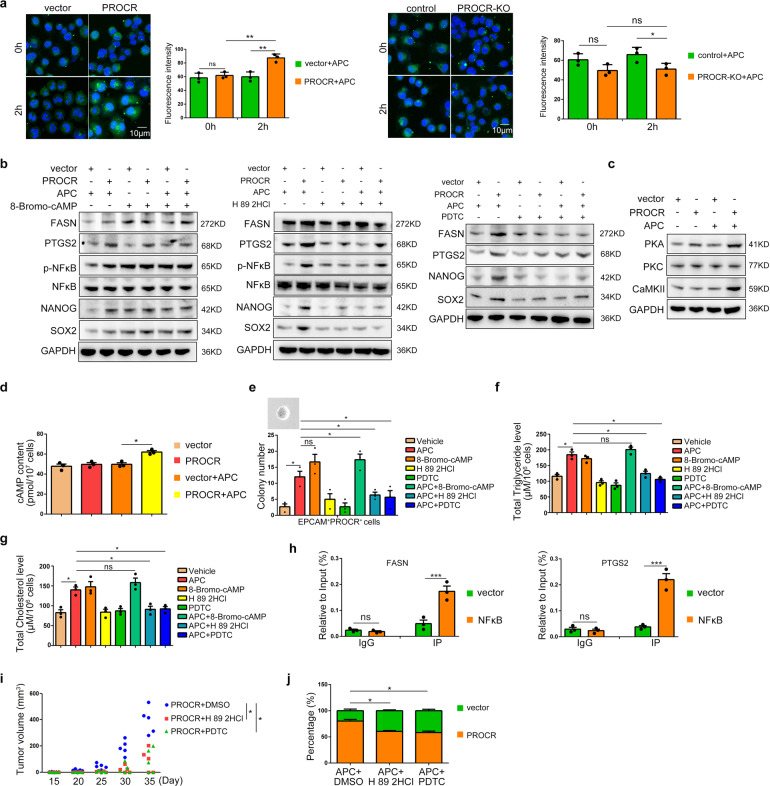
Signaling cascade induced by activated PROCR in NPC. **a** Ca^2+^ flux dynamics labeled by fluo-4 in *PROCR* overexpression or knockout cells. Living cells were observed right after the dye supplementation. The corresponding right panels show the statistical analysis of fluorescence intensity. **b**, **c** Western blotting analysis of lipid metabolism related genes expression and stem cell markers expression in vector or *PROCR* overexpressing cells with different drugs treatment. 8-Bromo-cAMP, PKA agonist; H 89 2HCl, PKA antagonist; PDTC, NFκB inhibitor. **d** ELISA detection of the cellular cAMP content in *PROCR* overexpressing cells. **e** Sphere forming assay of the sorted CD45^-^EPCAM^+^PROCR^+^ cells treated with different drugs. **f**, **g** The cell content of triglycerides and cholesterol in *PROCR* overexpressing cells treated with different drugs. **h** ChIP-PCR detection of the direct transcriptional regulation of NFκB on FASN or PTGS2 expression. **i** Subcutaneous xenograft tumor volume of *PROCR* overexpressing cells treated with different drugs; *n* = 5 for each group. All mice received APC activation. **j** Percentages of GFP^+^ cells from xenograft tumor nodules in the lungs of mice treated with different drugs. The drugs were administered every week; ns Not significant, *P < 0.05, ***P < 0.001

Next, we wondered how activated PROCR stimulated lipid metabolism in NPC. Based on previous findings, activated PROCR could alter cytosolic Ca^2+^ flux in the endothelium.^21^ We used a calcium indicator (fluo-4 AM) to reveal that the cytosolic Ca^2+^ flux increased upon *PROCR* overexpression, but decreased in *PROCR* knockout cells (Fig. 5a). We then introduced the Ca^2+^ ATPase antagonist Thapsigargin for cell treatment, which facilitates Ca^2+^ release into the cytoplasm from the endoplasmic reticular (ER).^29^ Thapsigargin elevated cytosolic Ca^2+^ levels efficiently, even in *PROCR* knockout cells (Supplementary Fig. 6a). In addition, the expression of lipid metabolism related genes and stem cell related genes increased in *PROCR* knockout cells upon Thapsigargin treatment (Supplementary Fig. 6b, c,). Moreover, the cellular contents of triglycerides and cholesterol were elevated (Supplementary Fig. 6d, e), suggesting that Ca^2+^ signaling functions downstream of *PROCR* in the regulation of lipid metabolism in NPC.

The release of Ca^2+^ from the ER is tightly orchestrated by the cAMP-PKA signaling cascade.^30^ Therefore, we determined the dynamics of cAMP levels and PKA expression after manipulating *PROCR* expression. The results showed that both of them increased upon *PROCR* overexpression (Fig. 5c, d). Moreover, cytosolic Ca^2+^ flux was altered significantly when the cells were treated with a PKA agonist or antagonist (Supplementary Fig. 6f). Meanwhile, activation or repression of PKA resulted in similar phenotypes compared to manipulating *PROCR* expression, such as disturbed lipid metabolism and stem cell properties both in primary cells and in cell lines (Fig. 5e, f, Supplementary Fig. 6g), indicating that cAMP-PKA induced Ca^2+^ signaling recapitulated the function of PROCR in NPC. Moreover, cAMP agonist Forskolin and calcium chelator BAPTA-AM were also used for cell treatment. The results showed that forskolin facilitated Ca^2+^ release significantly, and promoted cell colony formation and FASH/PTGS2 protein expression, while blocking Ca^2+^ using BAPTA-AM exhibited the opposite effect (Supplementary Fig. 6h–j). In addition, our attempts to identify the direct downstream targets of Ca^2+^ in NPC focused on nuclear factor kappa B (NFκB), because we observed that NFκB phosphorylation was tightly regulated by PROCR mediated PKA activation and cytosolic Ca^2+^ flux (Fig. 5b, Supplementary Fig. 7a). The entry of phosphorylated NFκB into the nucleus was also detected after PROCR activation or Ca^2+^ release (Supplementary Fig. 7a). Inhibition of NFκB reduced the triglycerides and cholesterol contents in *PROCR* GOF cells (Fig. 5e, f). To determine whether activated NFκB directly regulates the transcription of lipid metabolism related genes, we first screened NFκB binding sites in the promotor regions of *FASN* or *PTGS2*, and cloned the identified sequences, with or without candidate site mutations, into the luciferase system. Together with the data from a chromatin immunoprecipitation (ChIP) experiment, the luciferase assay results confirmed the direct transcriptional regulation of NFκB on *FASN* and *PTGS2* (Supplementary Fig. 7b, c, Fig. 5h). Moreover, blockage of PKA or NFκB also inhibited subcutaneous tumor growth and femur xenograft metastasis in vivo (Fig. 5i, j), further confirming the orchestrated signaling cascade triggered by PROCR.

### PROCR serves as an important prognostic factor in NPC

**Fig. 6:**
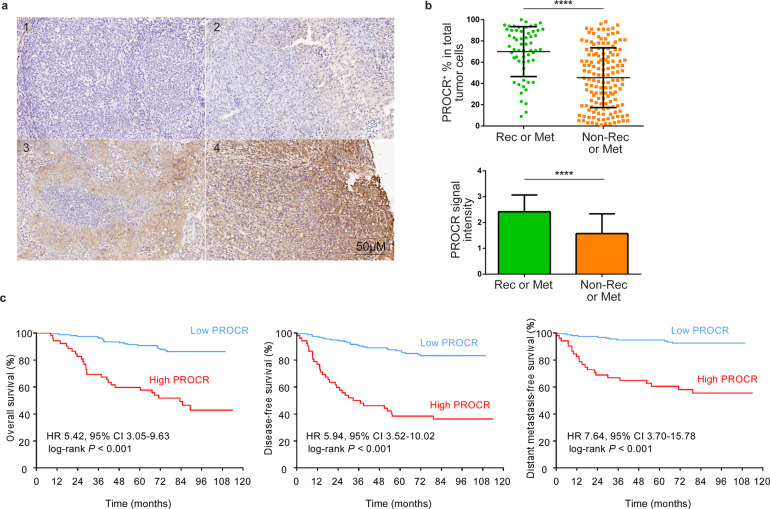
PROCR serves as an important prognostic factor in NPC. **a** PROCR expression in sectioned NPC biopsy samples embedded in paraffin. According to the staining intensity, a total of 207 samples were divided into four groups denoted by 0 to 3. **b** Quantification of the PROCR expression level in metastatic or recurrent patients (*n* = 58) versus non - metastatic or non-recurrent patients (*n* = 149) based on IHC staining. The upper panel shows the proportion of PROCR^+^ tumor cells versus total tumor cells in each sample in different groups. The lower panel shows the average staining intensity of PROCR in different sample groups. ****P < 0.0001. **c** Kaplan–Meier curves for patients’ overall survival, disease-free survival, and distant metastasis-free survival according to PROCR expression (PROCR^+^ cells %). X-tile software was used to generate the optimal cut-off value to stratify patients at different risks. In total, 155 (74.9%) patients had a low PROCR, while 52 (25.1%) patients had a high PROCR

Finally, to identify the prognostic value of PROCR in the clinic, we next examined the expression of PROCR in a cohort of 207 NPC specimens by immunohistochemistry (IHC). All of the samples were divided into four subgroups according to PROCR expression level (Fig. 6a). Moreover, we also evaluated the proportion of PROCR^+^ cells versus total tumor cells in each sample. We found that PROCR expression correlated with the TNM stages (Pearson, *r* = 0.4426, *p* < 0.0001), validating our flow cytometric results. In addition, it was revealed that both PROCR^+^ cell percentage and PROCR expression level increased significantly in metastatic or recurrent NPC patients (Fig. 6b), supporting our findings that PROCR^+^ cells displayed stemness potential. Prognostic analysis indicated that high PROCR expression predicted poor overall survival, disease-free survival, and distant metastasis-free survival (Fig. 6c). All of these data demonstrated that PROCR served as an important prognostic factor in the clinic.

## Discussion

Collectively, our findings identified a potential NPC CSCs labeled as CD45^-^EPCAM^+^PROCR^+^, which was capable of initiating primary or distant tumorous lesions in mice. Functional analysis revealed that PROCR is important for the acquisition and maintenance of cellular stemness potential in NPC. Mechanistic studies showed that activated PROCR signaled through cAMP-PKA-mediated Ca^2+^ flux to transmit stimuli into the nucleus, where NFκB enhanced lipid metabolism related gene transcription. Afterward, the activated lipid metabolism promoted mitochondrial fission, which potentiated the stemness potential of the NPC cells (Supplementary Fig. 8). However, how PROCR activates cAMP-PKA and whether PAR1 takes part in the regulation are still unknown, which needs to be further studied.

In our work, the canonical subcutaneous PDX model using chopped tumor tissues or sorted CD45^-^EPCAM^+^PROCR^+^ tumor cells were testified initially, while none of the recipient mice developed xenograft tumor. Inspired by the fact that most metastatic NPC patients developed bone metastasis, we believed the mice bone marrow might provide a suitable niche for disseminating tumor cells, like metastatic CSCs. In the long run, our PDX model suitable for tumor metastasis observation may facilitate the following drug screening or targeted therapies evaluation during individualized treatment in the clinic. In addition, we also found that the tumor promoting effect of PROCR was dependent on exogeneous APC treatment to a great extent. As the precursor of APC, Protein C (PROC) is abundant in blood serum.^31^ Therefore, it can be inferred that PROC derived APC takes part in the stimulation of PROCR^+^ tumor cells, since these cells tend to lie adjacent to the blood vessel in tumor tissue. This implied that the niche environment was critical for PROCR^+^ cell behaviors and partially explained the phenotypic differences with or without APC administration in our study.

Based on our results, loss of PROCR did not alter lipid metabolism and stemness potential of NPC cells. We tend to believe that PROCR is only needed for maintaining the stemness potential of NPC cells. The effect of PROCR depends on its expression level, and that there exists a threshold of PROCR expression level correlating with cellular activity. Our IHC data using patient specimens also support this, as higher PROCR expression correlates with advanced TNM stages or higher risk of metastasis or recurrence. Cancer cells are supposed to be hierarchically organized, from which only CSCs contribute to the maintenance and long-term tumor growth.^32^ Therefore, we think that PROCR^low/-^ cells are more likely the differentiated tumor cells, which might partially explain why the loss of PROCR did not show any observed effects. Moreover, considering the critical roles of lipid metabolism in cellular signaling transduction, bioenergy storage or even cell survival, we infer that there exists some unknown compensation mechanism to maintain the lipid synthesis at a normal level in response to PROCR loss of function. In this regard, we think that the activated lipid metabolism in tumor cells may not be PROCR specific, although their excessive activation is clearly induced by PROCR overexpression.

Mitochondrial fission and fusion are tightly regulated, which is beneficial for cells to maintain a homogenized mitochondria population, equitable inheritance of mitochondria and its DNA, and regular recycling of damaged mitochondria. The disorder of mitochondrial fission and fusion gave rise to cell malfunction in a series of diseases. It is believed that mitochondrial fission enables asymmetric partition of healthy and defective mitochondria during stem cell asymmetric division.^33,34^ Moreover, manipulating the expression of genes that are essential for fission or fusion influences on tumor progression and tumor initiation.^34–36^ Here, we revealed that hyperactive lipid synthesis induced by PROCR promotes mitochondrial fission. Despite of previous reports on lipid synthesis triggering mitochondrial fission in pluripotent stem cells,^26^ we speculate that lipid storage guarantees the extra energy or macromolecule demands for NPC CSCs during their metastasis or proliferation processes. Therefore, targeting lipid metabolism is regarded as a novel strategy against tumor cells, or even tumor stem cells.^15,16,37^ Our data uncovered that lipid synthesis abrogation suppressed CSCs self-renewal and metastasis. Theoretically, these findings provide novel clinical targets against metastatic or recurrent NPC.

## Materials and methods

### Patients and tumor tissue samples

Biopsy samples were obtained from patients with pathologically confirmed NPC (*n* = 51) at Sun Yat-sen University Cancer Center. A total of 212 paraffin embedded NPC specimens were retrospectively collected from Sun Yat-sen University Cancer Center. No patients received clinical treatment before sampling, and all patients provided written informed consent.

### Cell lines

The NPC cell lines HONE1 and 5–8 F were cultured in Roswell Park Memorial Institute (RPMI) 1640 medium (Invitrogen, Life Technologies, Grand Island, NY, USA) supplemented with 5% fetal bovine serum (FBS; Gibco, Carlsbad, CA, USA). To establish stable cell lines, the coding sequence of *PROCR* was cloned into vector pSin-EF2-puro. Stable overexpression cell lines were obtained by retroviral transduction and puromycin screening, and were confirmed by western blotting. For CRISPR-mediated gene knockout, the genomic guide RNA (gRNA: 5’-GTACCAGGGCAACGCGTCGC-3’) were searched online (https://zlab.bio/guide-design-resources) and cloned into lentiCRISPRv2 vector. The constructs were retrovirally transfected into NPC cells, followed by puromycin screening. The surviving cells were confirmed using western blotting.

### Flow cytometry

For the NPC biopsy samples, the tissues were digested into a single cell solution using collagenase IV (17104019, Invitrogen). The antibodies used in flow cytometry were as follows: anti-CD45 (304026, Biolegend, San Diego, CA, USA), anti-EPCAM (ab112068, Abcam, Cambridge, MA, USA), anti-PROCR (17-2018-41, Invitrogen), anti-NANOG (4903 S, Cell Signaling Technology, Danvers, MA, USA), and anti-SOX2 (3579 S, Cell Signaling Technology). The cells were analyzed using the cytoFLEX S system (Beckman, Indianapolis, IN, USA) and sorted using MoFlo Astrios (Beckman).

### Sphere formation assay

The sphere forming medium comprised Dulbecco’s modified Eagle’s medium (DMEM)/F-12 medium (11320082, Gibco, Thermo Fisher Scientific) with 20 ng/ml of Epidermal Growth Factor (EGF, RP-8661, Thermo Fisher Scientific, Waltham, MA, USA), 10 ng/ml of basic Fibroblast Growth Factor (bFGF, PHG0266, Gibco, Thermo Fisher Scientific), 5 μg/ml of Insulin, 0.4% Bovine serum albumin (BSA, 9048-46-8, Sigma-Aldrich, St. Louis, MO, USA), GLUTAMAX I (35050061, GIBCO), HEPES (15630080, Gibco) 1 × B-27 (17504044, Thermo Fisher Scientific) and Penicillin-Streptomycin (15070063, Gibco). About 1000 tumor cells were cultured in a 96-well ultra-low attachment microplate (4442, Corning, NY, USA). A new medium was supplemented every 3 days, and the colonies were observed and counted 4–7 days later, depending on the cells’ growth status. For sorted cells from specimens, a sphere diameter greater than or equal to 30 µm was considered effective, while for cell lines, the threshold was 50 µm.

### Drug treatment

For cell culture, the drugs treatment dosages were as follows: APC (100 nM, 55-3-08 A, Pepmic, Suzhou, China), PKA agonist (8-Bromo-cAMP, 10 μM, S7857, Selleck, Houston, TX, USA), PKA antagonist (H 89 2HCl, 10 μM, S1582, Selleck), NFκB inhibitor PDTC (Pyrrolidinedithiocarbamate ammonium, 10 μM, S3633, Selleck), Ca^2+^-ATPase inhibitor (Thapsigargin, 1 μM, ab120286, Abcam), FASN inhibitor (C75, 50 μM, S8915, Selleck), PTGS1 and PTGS2 inhibitor (Ketoprofen, 50 μM, S1645, Selleck), cAMP agonist (Forskolin, 10 μM, S2449, Selleck) and calcium chelator (BAPTA-AM, 10 μM, HY-100545, MedChemExpress). For in vivo treatment in mice, the dosage of drugs was: APC (300 μmol per mouse), H 89 2HCl (50 mg/kg), PDTC (50 mg/kg), C75 (30 mg/kg), and Ketoprofen (50 mg/kg). All of the drugs were diluted in phosphate-buffered saline (PBS) solution and injected into mice via the tail vein every week.

### RNA-sequencing

Total RNA was extracted using Trizol. The mRNA was purified from the extracted total RNA using OligodT and DNA probes to eliminate contamination from other nucleic acids. The mRNA was then fragmented and reverse transcribed into cDNA. A-Tailing Mix and RNA Index Adapters were added onto the cDNA, followed by PCR amplification. The quality of the constructed libraries was then assessed using an Agilent 2100 Bioanalyzer (Thermo Fisher Scientific) and the StepOnePlus Real-Time PCR System (ABI, Foster City, CA, USA). The high-quality libraries were sent for high-throughput sequencing using the Illumina HiSeq4000 platform (Illumina San Diego, CA, USA) by the Beijing Genomics Institute (BGI, Shenzhen, China). The clean reads were obtained after filtering the low-quality or contaminated raw reads. The reads were then queried against the target genome sequences (GCF_000001405.37_GRCh38.p11) using Bowtie2, and the genes expression levels were evaluated using RSEM (BGI).

### Lipidomics

Vector control and PROCR GOF cells were harvested after APC treatment. Each group contained four samples repeat. The lipidomics analysis was performed in BGI corporation. The analysis process of lipidomics is divided into experimental and bioinformatics analysis. The experimental process includes lipids extraction, LC-MS/MS detection, and so on. Bioinformatics analysis mainly includes: data preprocessing, data quality control, statistical analysis, screening of differential lipid molecules, etc. Multivariate statistical analysis and univariate analysis were used to screen different lipids between groups. Fold change analysis (FC analysis) and *T*-test were performed on the data. Fold change (FC) was obtained by FC analysis, *p*-value was obtained by *T*-test, and t.test_p.value_BHcorrect (qvalue) was obtained by FDR (False Discovery Rate) correction.

### Western blotting and immunofluorescence

Total protein was extracted using radio-immunoprecipitation assay lysis buffer (RIPA; Beyotime, Shanghai, China). Proteins were separated using sodium dodecyl sulfate-polyacrylamide gel electrophoresis and transferred onto polyvinylidene difluoride membranes (Millipore, Billerica, MA, USA). The membranes were then incubated with primary antibodies at 4 °C overnight. The antibodies were listed as follows: PROCR (Proteintech, 67658-1-Ig, 1:1000), FASN (Proteintech, 10624-2-AP, 1:1000), PTGS2 (Proteintech, 12375-1-AP, 1:1000), SOX2 (Cell Signaling Technology, 3579 S, 1:500), NANOG (Cell Signaling Technology, 4093 S, 1:1000), GAPDH (Proteintech, 60004-1-Ig, 1:20000), PKA (Proteintech, 55388-1-AP, 1:1000), PKC (Proteintech, 21991-1-AP, 1:1000), Phospho-NFκB (Cell Signaling Technology, 3033 S, 1:1000), NFκB (Cell Signaling Technology, 8242 S, 1:1000), CaMKII (Proteintech, 12666-2-AP, 1:1000), DRP1 (Proteintech, 12957-1-AP, 1:1000), FIS1 (Proteintech, 10956-1-AP, 1:1000), MFN1 (Proteintech, 13798-1-AP, 1:1000), COX4I1 (Proteintech, 11242-1-AP, 1:1000), UQCRC1 (Proteintech, 21705-1-AP, 1:1000). After incubation with species-matched secondary antibodies, the immunoreactive proteins were detected using chemiluminescence in a gel imaging system (ChemiDoc MP Imaging System, Bio-Rad, Hercules, CA, USA). For immunofluorescence, the sectioned tissues or cultured cells were incubated with antibodies at 4 °C overnight, followed by reaction with the corresponding secondary fluorescent antibody (1:500, A-21206, A-21203, Invitrogen) and Hoechst staining (1:5000, H3570, Invitrogen). Images were captured using confocal microscopy (Olympus, Tokyo, Japan). For Ca^2+^ labeling, lipid droplet staining and mitochondria staining, fluo-4 AM (Thermo, F14201), Nile red (Thermo, N1142), and MitoTracker (Thermo, M22426) was used as the manufacturer’s instructions.

### ELISA, triglyceride, or cholesterol detection

About 2 × 10^5^ cells were seeded in a 60 mm dish, and APC or other drugs were supplemented into the medium 36 h later. After about 12–24 h of treatment, the cells were harvested for enzyme-linked immunosorbent assay (ELISA) detection according to protocols supplied by the cAMP ELISA kit (L00460, GenScript, Piscataway, NJ, USA) and a PGE2 Express ELISA Kit (500141, Cayman, Michigan, USA), or for triglyceride and cholesterol detection according to protocols supplied by the Triglyceride-Glo™ Assay (J3160, Promega, Madison, WI, USA) and the Cholesterol/Cholesterol Ester-Glo™ Assay (J3190, Promega).

### Chromatin immunoprecipitation (ChIP) assay

The ChIP assay was performed in HONE1 cells overexpressing NFκB, or in those transformed with the control vector, using a Pierce™ Agarose ChIP Kit (26156, Thermo Fisher Scientific) according to the manufacturer’s protocol. After immunoprecipitation using anti-NFκB antibodies (10745-1-AP, Proteintech, Rosemont, IL, USA) or IgG, real-time PCR was applied to examine the enrichment of DNA fragments in the binging sites of the FASN or PTGS2 promoter. The primers used were as follows: FASN forward: 5’-GCGGGTCCGTCCGTCCTT-3′, FASN reverse: 5’- TCCCGAGCCATCCCCGAC-3′. PTGS2 forward: 5’-ACGTGACTTCCTCGACCCT-3’, PTGS2 reverse: 5’-ATCGCCTTGGATGGGATA-3’. The percentage of binding DNA fragments was quantified relative to the input.

### Luciferase reporter assay

The wild-type and NFκB binding site-mutated *FASN* or *PTGS2* promoters (1500 base pairs upstream of transcription start site) were cloned into firefly luciferase-expression vector psiCHECK™ (Promega). For the luciferase reporter assay, cells were cotransfected with NFκB or control vector and *FASN* or *PTGS2* promoter reporter vectors. The luciferase activity was examined using the Dual-luciferase Reporter System (Promega) following the manufacturer’s instructions.

### Cell proliferation, invasion, and wound healing assays

The CCK-8 assay was used to detect cell proliferation. Cells (1 × 10^3^) were seeded in 96-well plates, incubated for 0–4 days, and stained using CCK-8 (Dojindo, Tokyo, Japan). The absorbance was determined at 450 nm using a spectrophotometer. With regard to DDP treatment, various dosages of DDP were added into the culture medium 24 h after cell seeding. A total of 72 h later, cell viability was evaluated using the CCK-8 assay. For the cell migration or invasion assay, 3 × 10^3^ cells were seeded in 24-well Transwell chambers (Corning). The medium was supplemented with 10% FBS and placed in the bottom chambers. After 14–18 h of culture, the chambers were collected and the cells on the lower surface of the chambers were fixed in methanol and stained with crystal violet for observation. For the wound healing assay, tumor cells grown to near confluence in 6-well plates were subjected to serum-free medium for 24 h of starvation. Linear wounds were created in the cell monolayers using a P-10 pipette tip, followed by an additional 24 h of starvation before observation.

### Immunohistochemistry

For tumor nodules in mice, the lungs were dissected and fixed using 10% methanal before embedding in paraffin. The samples were sectioned into 5-μm slices and mounted on slides. The slides were incubated at 4 °C overnight with antibodies against KI67 (1:200, ab15580, Abcam) or CK-7 (1:1000, 17513-1-AP, Proteintech). For NPC specimens, the slides were incubated by PROCR antibody (Proteintech, 67658-1-ig, 1:1000) at 4 °C overnight. Afterward, the sections were then incubated with a biotinylated secondary antibody bound to a horseradish peroxidase complex. The antibody was visualized by adding 3,3-diaminobenzidine, and the sections were counterstained with hematoxylin. The whole view of each slide was scanned using the ScanScope Aperio AT2 slide scanner (Leica Microsystems) at 400× magnification.

### Animal experiments

B-NDG mice (non-obese diabetes, severe combined immunodeficiency with double knockout of the interleukin-2 receptor gamma chain and protein kinase DNA-activated catalytic genes: NOD-*Prkdc*^*scid*^ *IL2rg*^*tm1*^/Bcgen) were purchased from Biocytogen Jiangsu Co., Ltd. (Jiangsu, China). Cell suspensions with a total volume of 20 µl (15 µl tumor cells and 5 µl matrigel) were injected into the mice femurs after anesthesia using isoflurane. About 300 µmol APC diluted in PBS was administrated every 2 weeks. The mice were observed regularly. Some of the recipient mice died within two months without xenograft tumors, and we speculated that this might result from bacterial or viral infection in the tumor tissues. Mice were sacrificed when they exhibited sustained body weight loss and lack of appetite. The lungs were fixed in Bouin solution with 70% picric acid, 5% glacial acetic acid, and 25% methanol. For computed tomography (CT) imaging, the leg bones of the mice were dissected and examined under an Inveon PET-CT (Siemens, Munich, Germany). For tumor cell lines, 1 × 10^5^ *PROCR* overexpressing or knockout cells with green fluorescent protein (GFP) expression were mixed with the same amount of their corresponding control cells, and were transplanted into the femurs. Five weeks later, the mice were sacrificed and the lungs were dissected for analysis. For the subcutaneous tumor xenograft model, a certain amount of tumor cells was diluted to 200 µl with PBS and matrigel. After transplantation, the subcutaneous tumor xenograft was evaluated and recorded regularly. Tumor volume was calculated as follows: volume = D × d^2^ × π/6, where D represents the longest diameter and d represents the shortest diameter of the tumor bulk. About 300 µmol APC diluted in PBS was injected into mice PB every week via the tail vein. All animal research was performed in accordance with the detailed rules approved by the Sun Yat-sen University Cancer Center Animal Care and Use Ethics Committee. All efforts were made to minimize animal suffering.

### Statistical analysis

Statistical analyses were performed using SPSS 17.0 (IBM Corp., Armonk, NY, USA). All in vitro data shown were representative of at least three independent experiments, and values are reported as the mean ± SD. Differences between the two groups were analyzed using the two-tailed unpaired Student’s *t*-test. The correlation analysis was performed using Spearman with a two-tailed test. *P* < 0.05 was considered significant. All data in our study have been recorded at Sun Yat-sen University Cancer Center for future reference (RDDB2021891406).

## Supplementary information

Supplementary Materials

## Data Availability

The additional data in this study are available upon reasonable request.
