# Supplementary material for: Protein C receptor maintains cancer stem cell properties via activating lipid synthesis in nasopharyngeal carcinoma
Source: Signal Transduct Target Ther. 2022 Feb 16;7:46. doi: 10.1038/s41392-021-00866-z (PMC8847456; doi:10.1038/s41392-021-00866-z)
Supplement: Supplementary file 1 — Supplementary Materials [file 41392_2021_866_MOESM1_ESM.docx]

Supplementary Materials for

Protein C receptor maintains cancer stem cell properties via activating lipid synthesis in nasopharyngeal carcinoma

Panpan Zhang^1^*, Qiuping He^2^*, Yaqin Wang^1^*, Guanqun Zhou^1^, Yupei Chen^1^, Linglong Tang^1^, Yuan Zhang^1^, Xiaohong Hong^1^, Yanping Mao^1^, Qingmei He^1^, Xiaojing Yang^1^, Na Liu^1#^, Jun Ma^1#^

Correspondence to: majun2@mail.sysu.edu.cn, liun1@sysucc.org.cn, zhangpp@sysucc.org.cn

**This PDF file includes:**

Figures. S1 to S8

Tables S1

Figure. S1


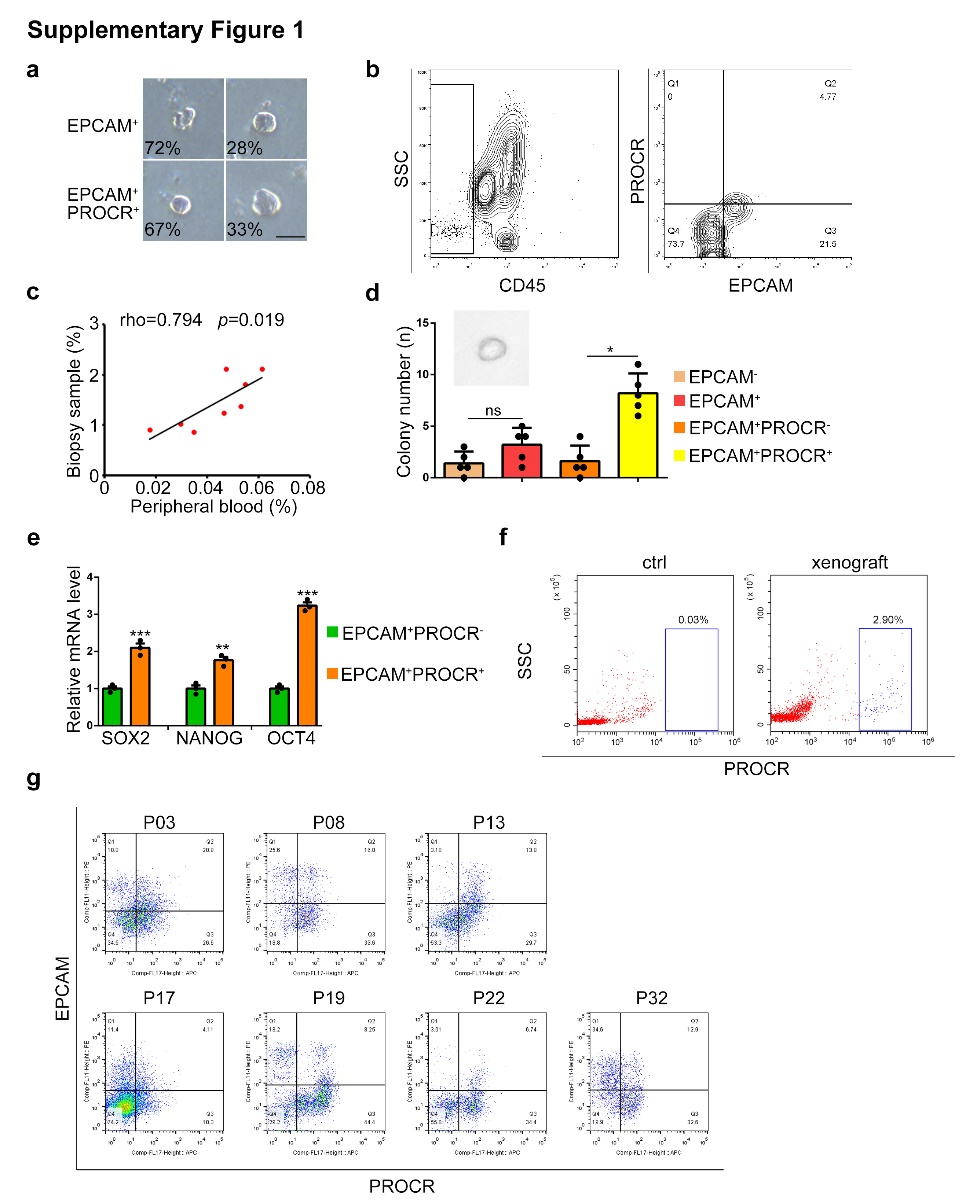


Figure S1. PROCR serves as a CSCs marker in NPC. (a) Various tumor spheres distribution formed by CD45-EPCAM+PROCR+ cells which were sorted from patient biopsy samples. The cells were incubated in the presence of APC. (b) Flow cytometry analysis of the circulating CD45-EPCAM+PROCR+ tumor cells in patients’ peripheral blood. Red blood cells were removed before antibodies staining. (c) Correlation analysis of the proportion of CD45-EPCAM+PROCR+ cells from the patients’ peripheral blood versus biopsy samples. n=8. Pearson analysis. (d) Sphere forming assay of the sorted CD45-EPCAM+PROCR+ cells from patients’ peripheral blood. The cells were incubated in the presence of APC. (e) mRNA expression level of stem cell marker genes in sorted CD45-EPCAM+PROCR+ cells by RT-qPCR. (f) Flow cytometry analysis of CD45-PROCR+ cells in peripheral blood from control or tumor xenograft mice. A total of 20-30µl blood cells were collected from wounded tail vein, and the recipient mice were sampled at least three months after tumor transplantation. (g) Flow cytometric analysis of patient biopsy samples which initiated xenograft tumor in mice. The cells were gated from CD45- population.

Figure. S2.


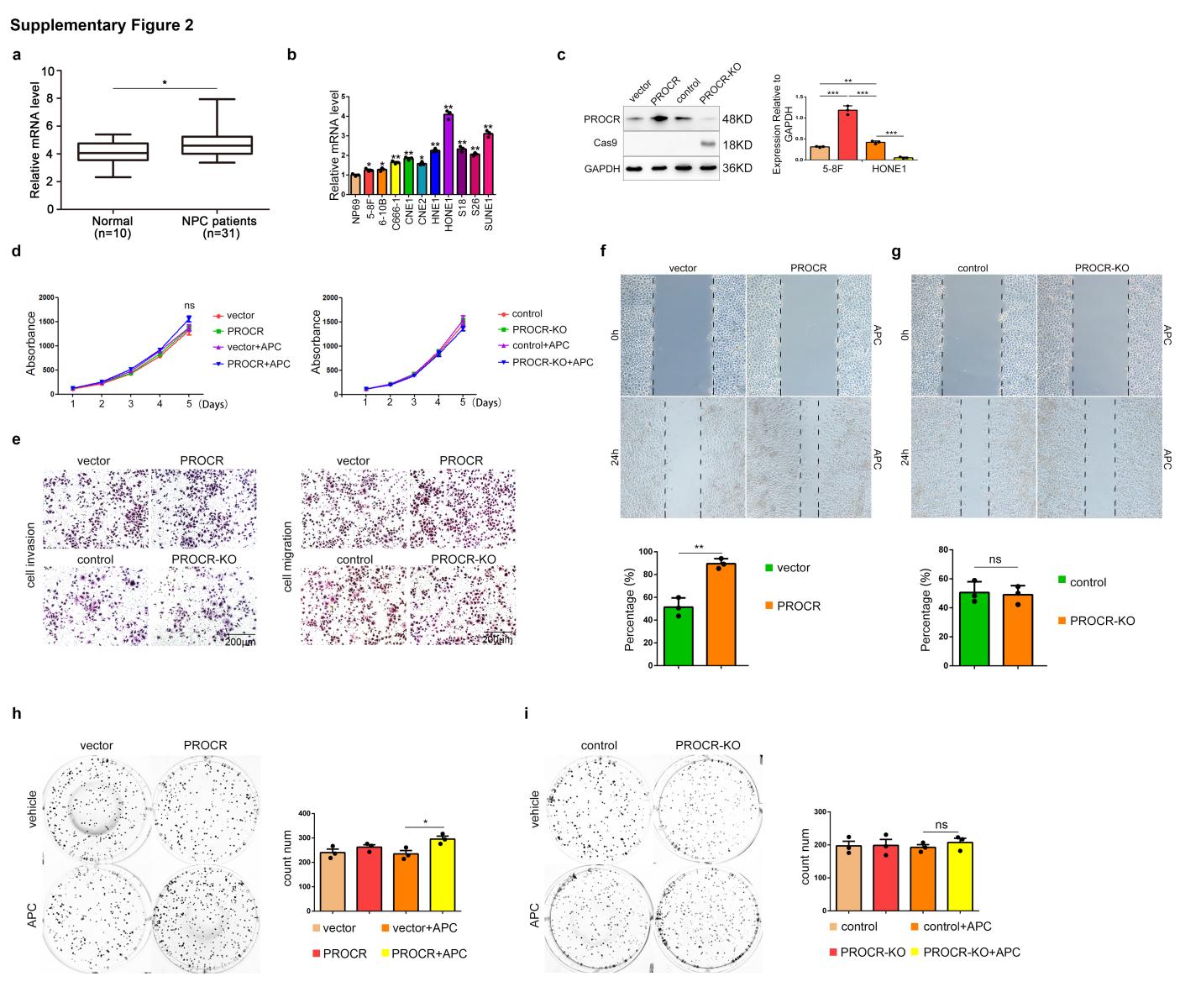


**Figure S2. Activated PROCR promotes tumor cell invasion and migration.** (a) Comparison of PROCR expression in healthy controls and patients with NPC using the GEO dataset (GSE81687280). (b) RT-qPCR detection of PROCR mRNA expression in various NPC cell lines. (c) Western blotting examination of PROCR protein levels in GOF or LOF cells. Right panel shows the quantification results. PROCR protein expression exhibited a 4-5 fold increase in GOF cells compared with vector counterpart, while exhibited a 3 fold increase compared with HONE1 cells. (d) CCK-8 assay of NPC cells with PROCR overexpression or knockout. (e) Cell invasion and migration assay of PROCR overexpressing or knockout cells. APC was present in the processes of cell culture. (f, g) Wound healing assay of PROCR overexpressing or knockout cells and their corresponding statistical analysis. (h, i) Cell replating assay of PROCR overexpressing or knockout cells, with or without APC treatment. ns, not significant, * P<0.05, ** P<0.01.

Figure. S3.


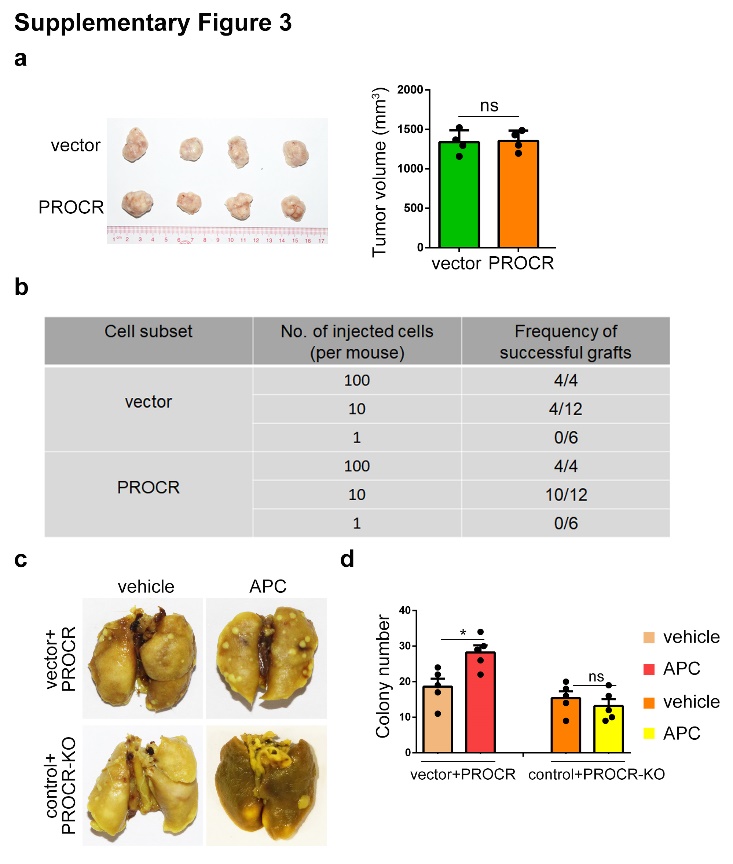


**Figure S3. PROCR overexpression strengthens the stemness potential of tumor cells.** (a) Subcutaneous xenograft tumor formed by vector or PROCR overexpressed NPC cells. The tumor volumes were quantified in the right panel. (b) Frequency of the successful induction of subcutaneous xenograft tumors, with or without PROCR overexpression. The CSC frequency is estimated to be 1 in 6.34 in PROCR GOF group versus 1 in 24.46 in vector control group according to Extreme Limiting Dilution Analysis (ELDA) with 0.95 confidence interval and a p-value of 0.0152. (c) Representative images showing nodules in recipient mice lungs with PROCR overexpression or PROCR knockout cells mixed with their counterpart control. (d) Quantification of the nodule number in each recipient mouse. n=3 for each group.

Figure. S4.


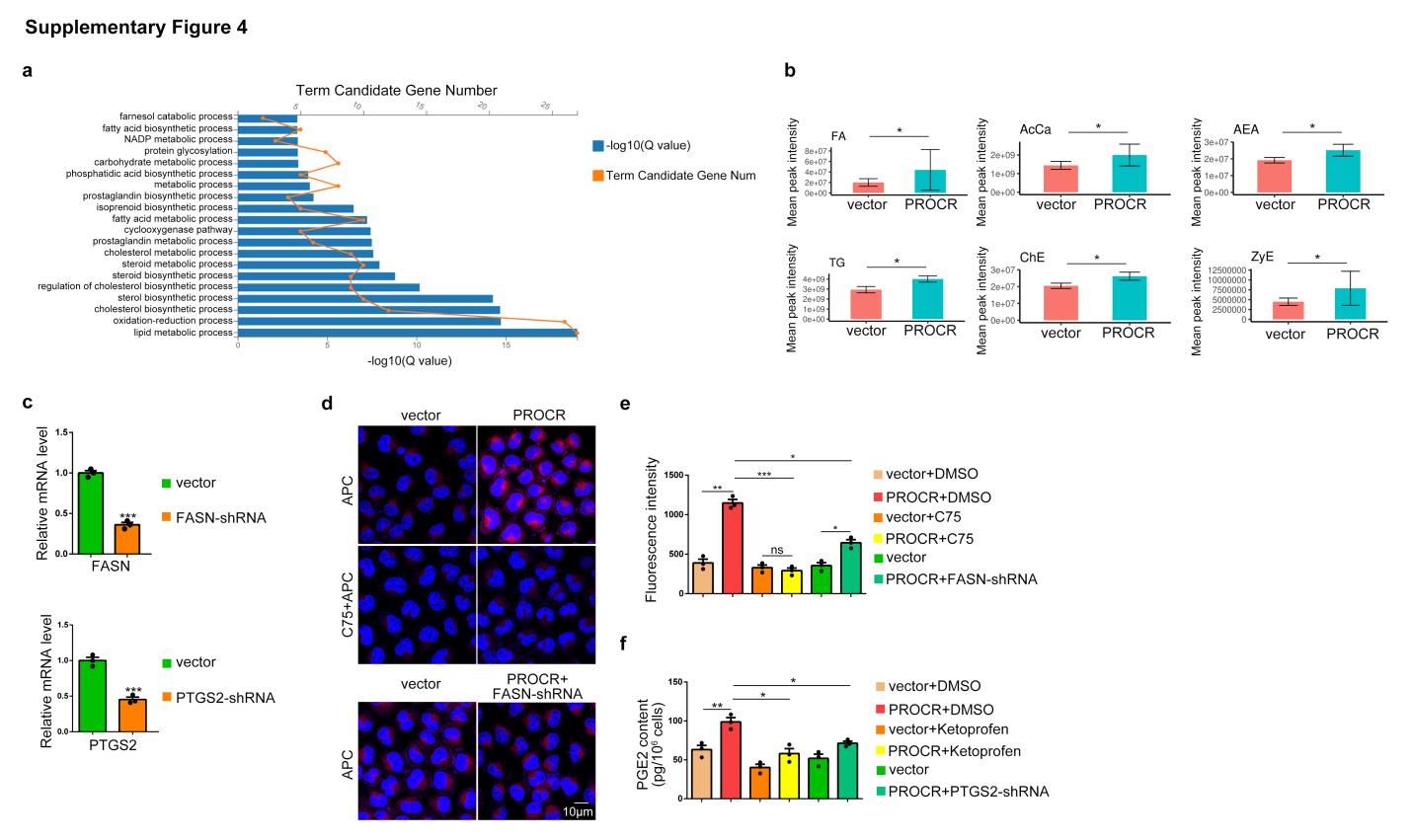


**Figure S4. Lipid metabolism is hyperactive in NPC cells upon PROCR activation.** (a) KEGG analysis of differentially expressed genes between vector control and PROCR overexpressing cells under conditions of APC activation. (b) Detected changes in lipid sub-class by lipidomics analysis. FA: Fatty acyls, AcCa: Fatty acyls, AEA: Fatty acyls, TG: Glycerolipids, ChE: Sterol Lipids, ZyE: Sterol Lipids. * P<0.05. (c) RT qPCR detection of FASN or PTGS2 mRNA expression level in cells with their corresponding shRNA knockdown. (d, e) Nile red staining of vector control or PROCR overexpressing cells treated with drugs or shRNA. The right panel shows the fluorescence intensity analysis. (f) ELISA analysis of PGE2 level in vector control or PROCR overexpressing cells treated with drugs or shRNA.

Figure. S5.


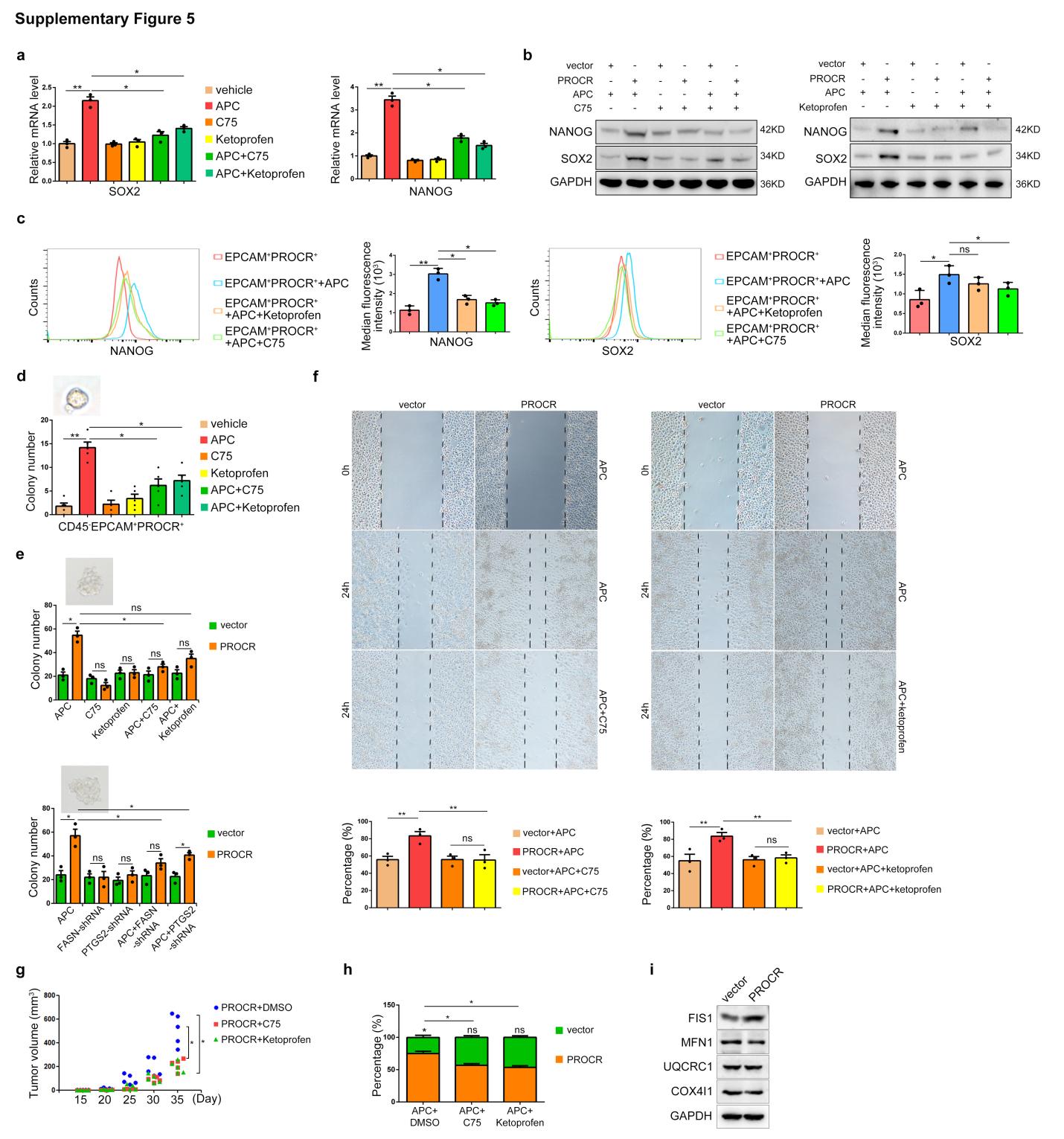


**Figure S5. Both fatty acids and PGE2 are indispensable for tumor cells to maintain CSC features.** (a) RT-qPCR detection of SOX2 and NANOG mRNA expression in PROCR overexpressing cells treated with different inhibitors. (b) Western blotting examination of vector control or PROCR overexpressing cells treated with different inhibitors. (c) Flow cytometry analysis of NANOG and SOX2 expression in PROCR overexpressing cells. Cells were harvested after 24 h of APC or inhibitors treatment. (d) Quantification of tumor spheres formed by sorted assay of CD45^-^EPCAM^+^PROCR^+^ cells in NPC biopsy samples with different drugs treatment. The sorted cells from three different patients were mixed together for sphere formation assay. (e) Sphere forming assay of vector control or PROCR overexpressing cells treated with different inhibitors or shRNAs. (f) Wound healing assay of vector control or PROCR overexpressing cells treated with different inhibitors. The bottom panels show their matching statistical analysis. (g) Subcutaneous xenograft tumor volume of PROCR overexpressing cells with different drug treatments; n = 5 for each group. All mice received APC activation regularly. (h) Percentages of GFP+ cells based on cytometric analysis from xenograft tumor nodules in mouse lungs under different drugs treatment. The drugs were administered every week. ns, not significant, * P<0.05, ** P<0.01. (i) Western blot detection of mitochondrial fission and fusion dynamics in vector or PROCR GOF cells. Cells were pretreated by APC.

Figure. S6.


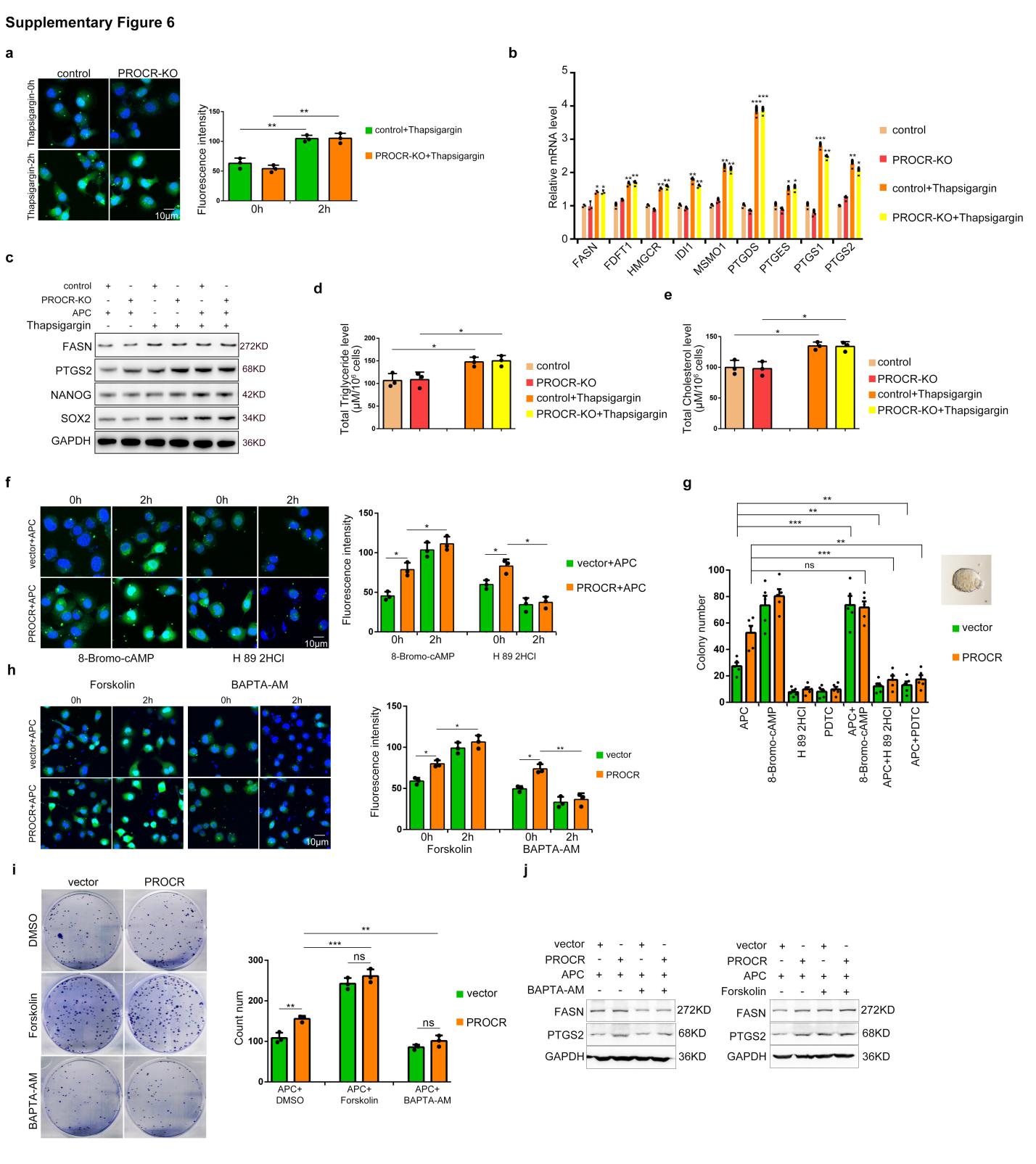
Figure S6. PROCR induces Ca2^+^ flux and the related signaling cascade activation. (a) Ca2+ indicator staining of control and PROCR knockout cells, with or without Thapsigargin treatment. Right panel shows the fluorescence intensity analysis. Thapsigargin was added into the culture medium 15 min before image capture. (b) RT-qPCR detection of various lipid metabolism-related genes in control or PROCR knockout cells. Cells were harvested 24h after Thapsigargin treatment. (c) Western blotting results of control or PROCR knockout cells with Thapsigargin treatment. (d, e) The cell content of triglycerides and cholesterol in PROCR overexpression or knockout cells. Cells were harvested 24h after Thapsigargin treatment. (f) Cytosolic Ca2+ dynamics in PROCR overexpressing cells treated with various drugs. The right panel denotes the fluorescence intensity analysis. (g) Sphere forming assay of cells with or without PROCR overexpression treated by different drugs. (h) Fluo-4 AM labelled calcium release in tumor cells before and 2 hours after drugs treatment. (i) cell plating assay of vector or PROCR GOF cells with different drugs treatment. (j) Western blot detection of FASN and PTGS2 expression in vector or PROCR GOF cells with different drugs treatment. The cells were harvested 24 hours after drugs treatment.

Figure. S7.


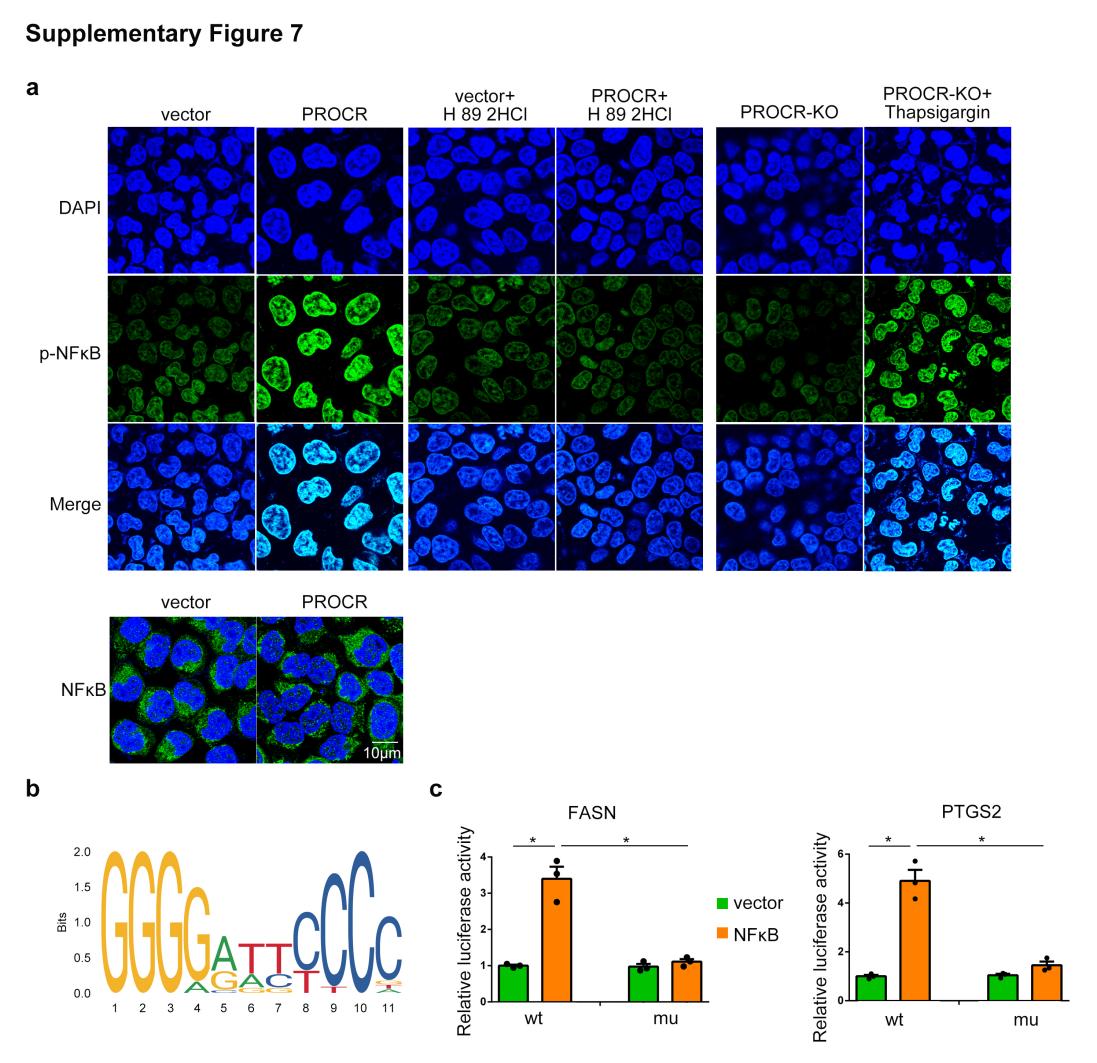


Figure S7. PROCR promotes NFκB activation to regulate lipid metabolism-related gene transcription. (a) Immunofluorescence staining of phosphorylated NFκB in PROCR overexpressing or knockout cells treated with various drugs. (b) Diagram showing NFκB binding sites. Predicted by JASPAR website. (c) Luciferase assay examining the direct regulation of NFκB on FASN and PTGS2. 2000 base pairs upstream of the transcriptional start site (TSS), with or without an NFκB binding site mutation, were cloned and transfected into cells with or without NFκB overexpression.

Figure. S8.


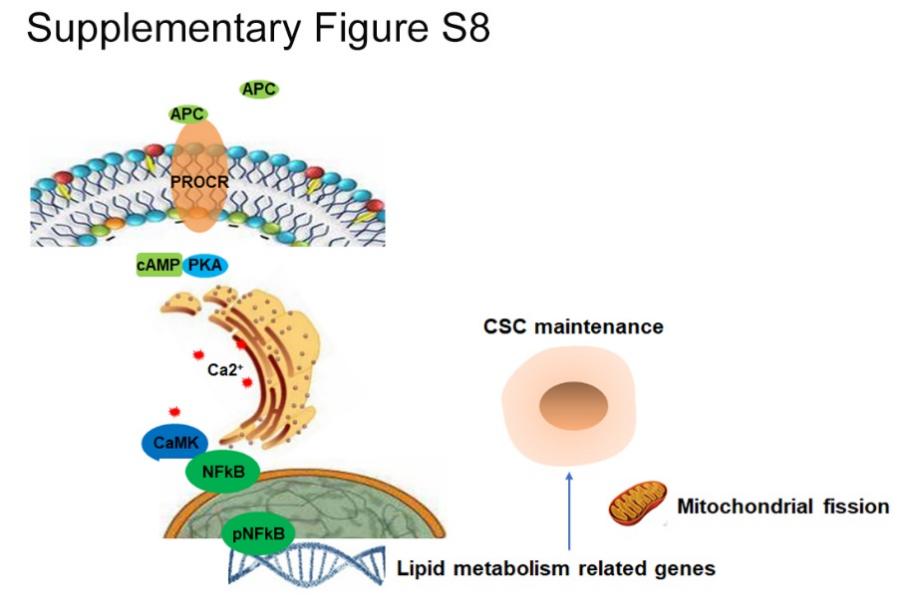


Figure S8. Working model of PROCR in nasopharyngeal carcinoma. Activated PROCR signals through cAMP-PKA to stimulate calcium release from endoplasmic reticulum. Afterwards, calcium mediated CaMK activates NFκB, and phosphorated NFκB triggers lipid metabolism related genes transcription. Sequentially, aberrant lipid synthesis and accumulation facilitate mitochondrial fission and cancer stem cell maintenance.

Table S1. The detailed information of patient samples used in the PDX experiments.

| Patient ID | Specimen | TNM staging | Total staging | CD45-EPCAM+PROCR+ cells (%) | cell type | successful engraftment (Y/N) |
| --- | --- | --- | --- | --- | --- | --- |
| P01 | tumor cell | T2N2 | III |  | NPC | N |
| P02 | tumor cell | T1N2 | III |  | NPC | N |
| P03 | tumor cell | T3N3 | IVA | 0.733 | NPC | N |
| P03 | tumor cell | T3N3 | IVA | 0.733 | CD45-EPCAM+PROCR+/- | Y |
| P04 | tumor cell | T4N1M1 | IVB | 0.267 | CD45-EPCAM+PROCR+/- | N |
| P05 | tumor cell | T2N0 | II | 0.417 | CD45-EPCAM+PROCR+/- | N |
| P06 | tumor cell | T4N2 | IVA | 0.837 | CD45-EPCAM+PROCR+/- | N |
| P07 | tumor cell | T4N1 | IVA | 2.459 | CD45-EPCAM+PROCR+/- | N |
| P08 | tumor cell | T3N3M1 | IVB | 2.021 | CD45-EPCAM+PROCR+/- | Y |
| P09 | tumor cell | T3N0 | III | 0.881 | CD45-EPCAM+PROCR+/- | N |
| P10 | tumor cell | T3N2 | III | 0.342 | CD45-EPCAM+PROCR+/- | N |
| P11 | tumor cell | T3N1 | III | 0.81 | CD45-EPCAM+PROCR+/- | N |
| P12 | tumor cell | T4N1 | IVA | 1.175 | CD45-EPCAM+PROCR+/- | N |
| P13 | tumor cell | T3N2M1 | IVB | 1.505 | CD45-EPCAM+PROCR+/- | Y |
| P14 | tumor cell | T3N1 | III | 0.697 | CD45-EPCAM+PROCR+/- | N |
| P15 | tumor cell | T4N1 | IVA | 0.928 | CD45-EPCAM+PROCR+/- | N |
| P16 | tumor cell | T3N1 | III | 0.423 | CD45-EPCAM+PROCR+/- | N |
| P17 | tumor cell | T3N1 | III | 0.332 | CD45-EPCAM+PROCR+/- | Y |
| P18 | tumor cell | T3N2 | III | 0.044 | CD45-EPCAM+PROCR+/- | N |
| P19 | tumor cell | T3N3 | IVA | 1.966 | CD45-EPCAM+PROCR+/- | Y |
| P20 | tumor cell | T2N2 | III | 0.671 | CD45-EPCAM+PROCR+/- | N |
| P21 | tumor cell | T3N0 | III | 0.166 | CD45-EPCAM+PROCR+/- | N |
| P22 | blood | T2N3 | IVA | 0.372 | CD45-EPCAM+PROCR+/- | Y |
| P23 | blood | T4N1 | IVA | 0.205 | CD45-EPCAM+PROCR+/- | N |
| P24 | tumor cell | T3N0 | III | 0.213 | CD45-EPCAM+PROCR+/- | N |
| P25 | tumor cell | T3N2 | III | 1.7 | CD45-EPCAM+PROCR+/- | N |
| P26 | tumor cell | T3N3 | IVA | 0.993 | CD45-EPCAM+PROCR+/- | N |
| P27 | tumor cell | T3N2 | III | 0.731 | CD45-EPCAM+PROCR+/- | N |
| P28 | blood | T3N2 | III | 0.217 | CD45-EPCAM+PROCR+/- | N |
| P29 | tumor cell | T3N1 | III | 0.163 | CD45-EPCAM+PROCR+/- | N |
| P30 | tumor cell | T3N3 | IVA | 0.748 | CD45-EPCAM+PROCR+/- | N |
| P31 | tumor cell | T3N1 | III | 0.649 | CD45-EPCAM+PROCR+/- | N |
| P32 | tumor cell | T3N2M1 | IVB | 1.244 | CD45-EPCAM+PROCR+/- | Y |
| P33 | tumor cell | T2N1 | II | 0.286 | CD45-EPCAM+PROCR+/- | N |
